# Supplementary material for: Bacteria, Fungi, and Protists Exhibit Distinct Responses to Managed Vegetation Restoration in the Karst Region
Source: Microorganisms. 2024 May 26;12(6):1074. doi: 10.3390/microorganisms12061074 (PMC11205577; doi:10.3390/microorganisms12061074)
Supplement: Supplementary file 1 [file microorganisms-12-01074-s001.zip › microorganisms-2999229-supplementary.pdf]

Additional file for:

Bacteria, Fungi, and Protists Exhibit Distinct Responses to Managed Vegetation Restoration in the Karst Region

Can Xiao [1,2,3], Dan Xiao [1*], Mingming Sun [1,2,4*], and Kelin Wang [2*]

[1] Key Laboratory of Agro-ecological Processes in Subtropical Region, Institute of Subtropical Agriculture, Chinese Academy of Sciences, Changsha 410125, China

[2] Huanjiang Observation and Research Station for Karst Ecosystems, Chinese Academy of Sciences, Huanjiang 547100, China

[3] College of Environment and Ecology, Hunan Agricultural University, Changsha 410128, China; sx20200047@stu.hunau.edu.cn (Z.S.)

[4] University of Chinese Academy of Sciences, Beijing 100039, China

[*]Corresponding authors:

Dan Xiao (E-mail: danxiao@isa.ac.cn; Tel: 86-0731-84619720; Fax: 86-0731-84612685); **Kelin Wang** (E-mail: kelin@isa.ac.cn; Tel: 86-0731-84615201; Fax: 86-731-84612685)

List of additional Figures

Figure S1 Soil physical and chemical properties of different vegetation restoration types.
CR, PF, FG, and FF represent cropland, planted forests, forage grass, and a mixture of plantation forest and forage grass, respectively. Significant differences ($p < 0.05$) among these vegetation restoration types are denoted by lowercase letters.

Figure S2 Relative abundance of bacterial, fungal, and protistan taxa at the genus level.
CR, PF, FG, and FF represent cropland, planted forests, forage grass, and a mixture of plantation forest and forage grass, respectively. Significant differences ($p < 0.05$) among these vegetation restoration types are denoted by lowercase letters.

Figure S3 Pearson's correlation revealing relationships among the genus-level abundance of bacteria, fungi, and protist.
Circle size represents correlation strength, with larger circles indicating stronger correlations. Significant correlation is indicated by asterisks (*$p < 0.05$; *$p < 0.01$).

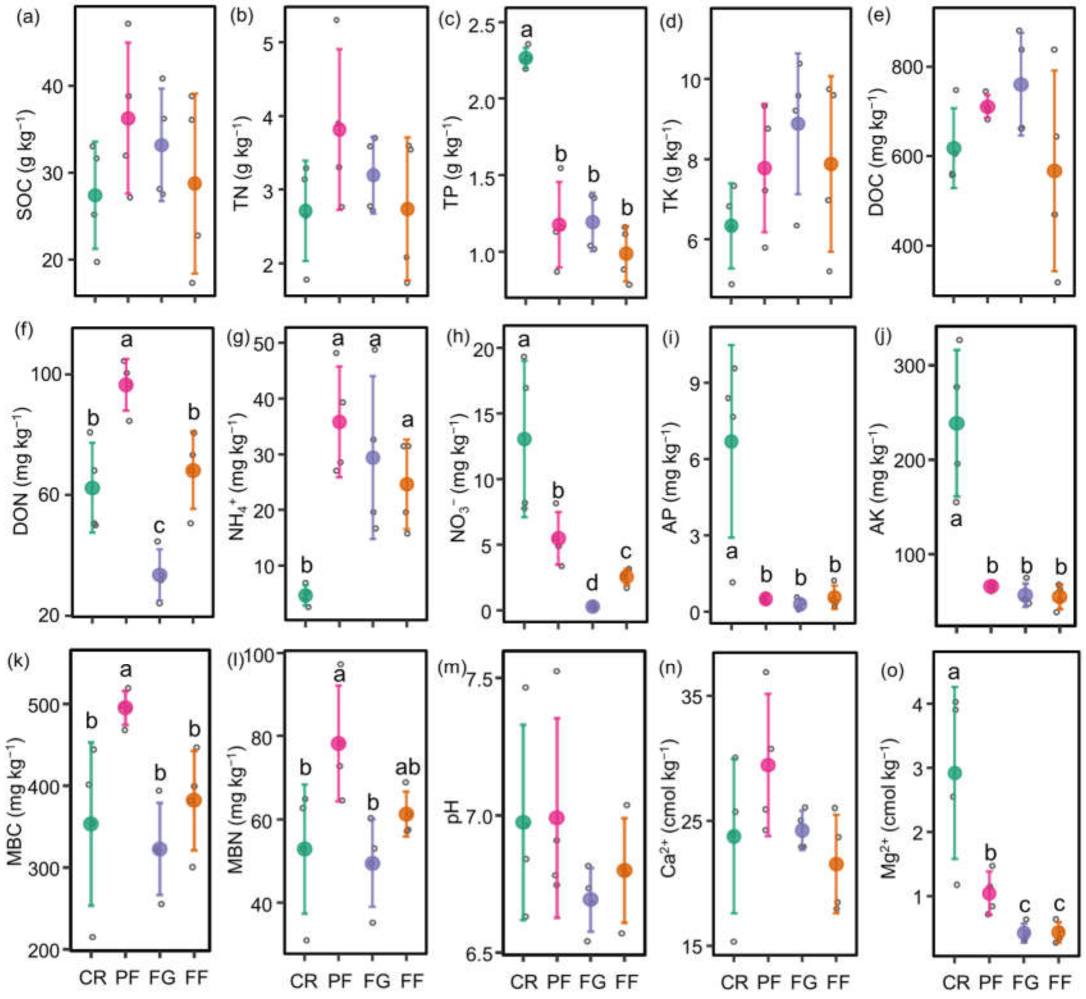

Figure S1 Soil physical and chemical properties of different vegetation restoration types.

CR, PF, FG, and FF represent cropland, planted forests, forage grass, and a mixture of plantation forest and forage grass, respectively. Significant differences ($p < 0.05$) among these vegetation restoration types are denoted by lowercase letters.

oil physicochemical

Note, soil physical and chemical properties used in this study was cited from our previous study by Sun et al., (2024).

Sun, M.; Xiao, D.; Zhang, W.; Wang, K. (2024). Impacts of managed vegetation restoration on arbuscular mycorrhizal fungi and diazotrophs in karst ecosystems. *Journal of Fungi* **2024**, 10(4), 280.

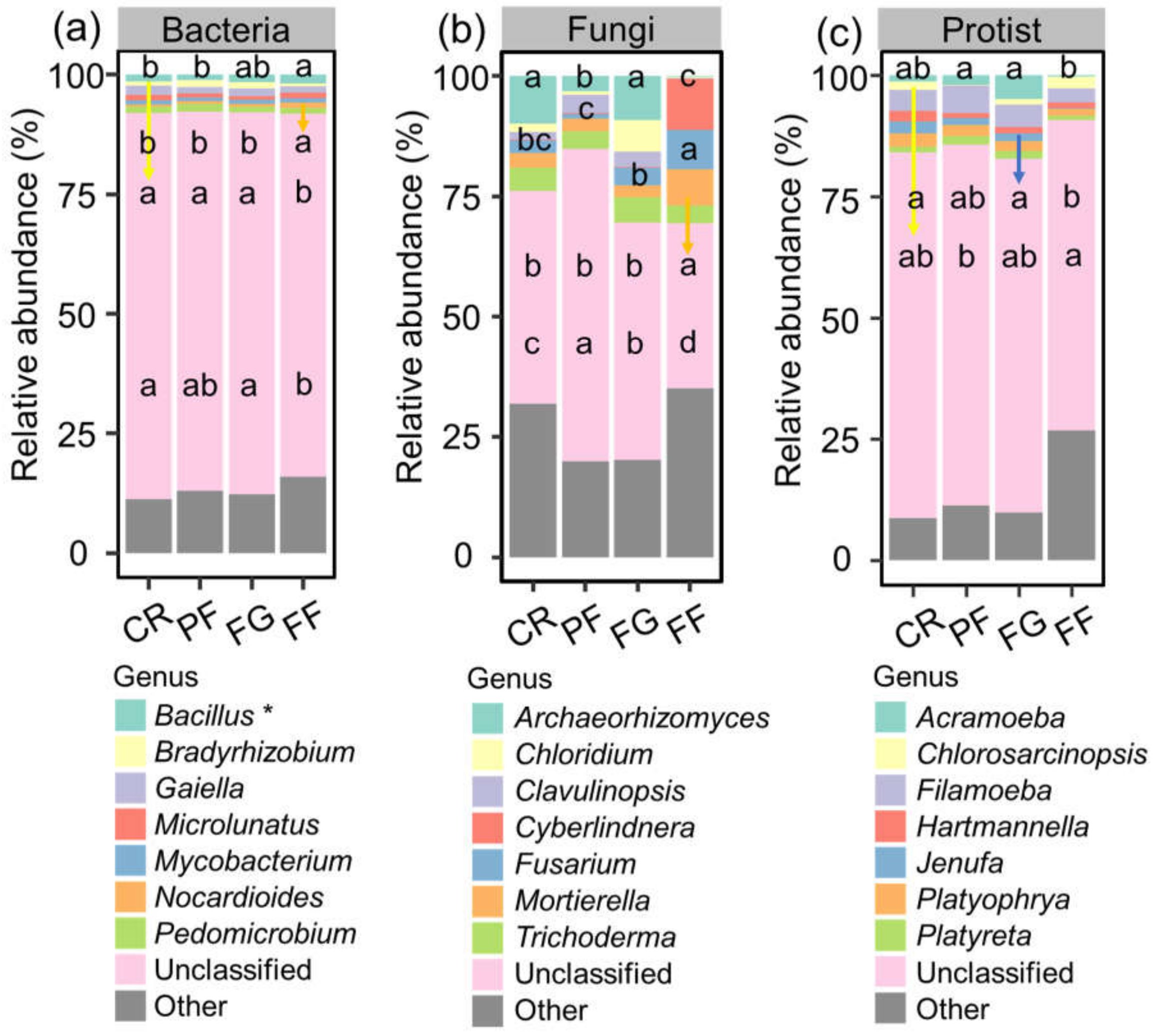

Figure S2 Relative abundance of bacterial, fungal, and protistan taxa at the genus level.

CR, PF, FG, and FF represent cropland, planted forests, forage grass, and a mixture of plantation forest and forage grass, respectively. Significant differences ($p < 0.05$) among these vegetation restoration types are denoted by lowercase letters.

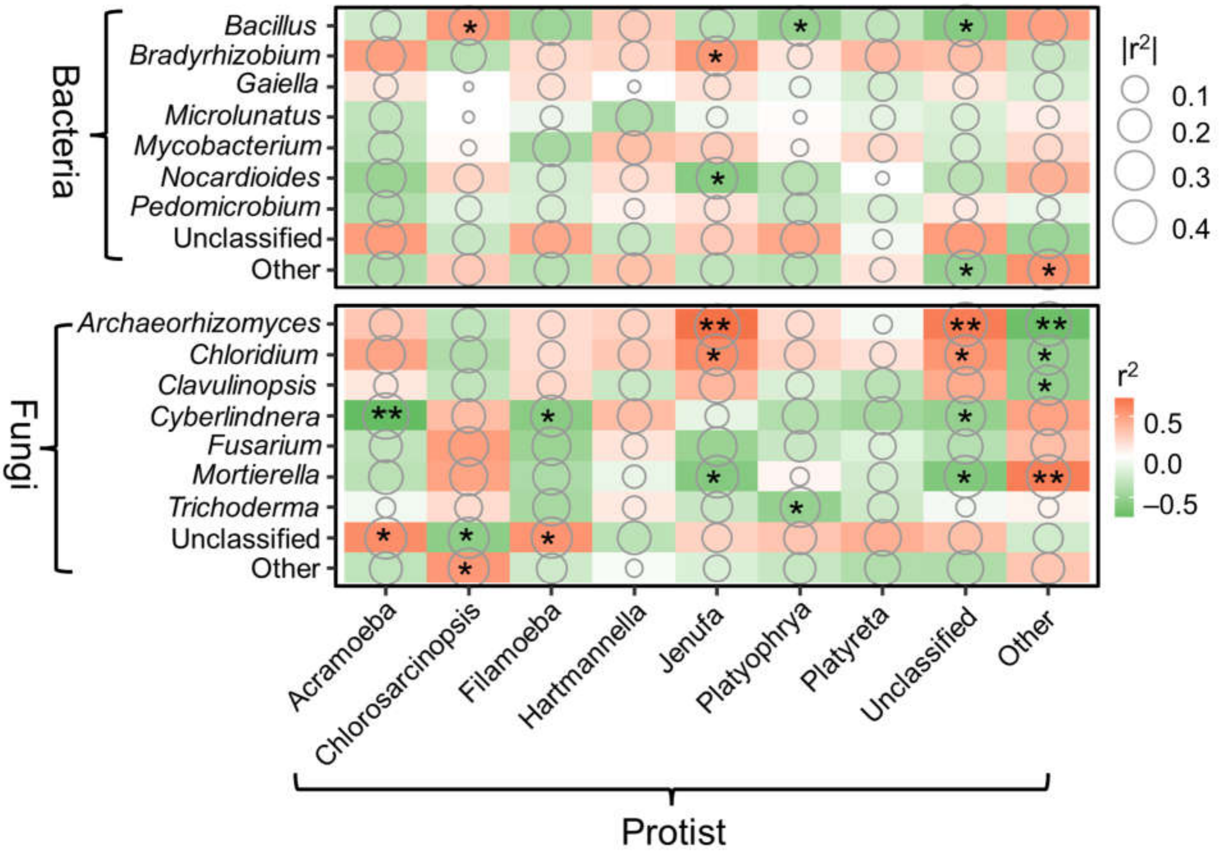

Figure S3 Pearson's correlation revealing relationships among the genus-level abundance of bacteria, fungi, and protist.

Circle size represents correlation strength, with larger circles indicating stronger correlations. Significant correlation is indicated by asterisks (*$p < 0.05$; *$p < 0.01$).
